# Increases in Mitochondrial DNA Content and 4977-bp Deletion upon ATM/Chk2 Checkpoint Activation in HeLa Cells

**DOI:** 10.1371/journal.pone.0040572

**Published:** 2012-07-10

**Authors:** Rong Niu, Minoru Yoshida, Feng Ling

**Affiliations:** Chemical Genetics Laboratory, RIKEN Advanced Science Institute, and CREST, JST, Wako-shi, Saitama, Japan; University of Texas Health Science Center at San Antonio, United States of America

## Abstract

Activation of the Mec1/Rad53 damage checkpoint pathway influences mitochondrial DNA (mtDNA) content and point mutagenesis in *Saccharomyces cerevisiae*. The effects of this conserved checkpoint pathway on mitochondrial genomes in human cells remain largely unknown. Here, we report that knockdown of the human DNA helicase RRM3 enhances phosphorylation of the cell cycle arrest kinase Chk2, indicating activation of the checkpoint via the ATM/Chk2 pathway, and increases mtDNA content independently of TFAM, a regulator of mtDNA copy number. Cell-cycle arrest did not have a consistent effect on mtDNA level: knockdown of cell cycle regulators PLK1 (polo-like kinase), MCM2, or MCM3 gave rise, respectively, to decreased, increased, or almost unchanged mtDNA levels. Therefore, we concluded that the mtDNA content increase upon RRM3 knockdown is not a response to delay of cell cycle progression. Also, we observed that RRM3 knockdown increased the levels of reactive oxygen species (ROS); two ROS scavengers, N-acetyl cysteine and vitamin C, suppressed the mtDNA content increase. On the other hand, in RRM3 knockdown cells, we detected an increase in the frequency of the common 4977-bp mtDNA deletion, a major mtDNA deletion that can be induced by abnormal ROS generation, and is associated with a decline in mitochondrial genome integrity, aging, and various mtDNA-related disorders in humans. These results suggest that increase of the mitochondrial genome by TFAM-independent mtDNA replication is connected, via oxidative stress, with the ATM/Chk2 checkpoint activation in response to DNA damage, and is accompanied by generation of the common 4977-bp deletion.

## Introduction

Human mitochondrial DNA (mtDNA) consists of a 16.5-kb circular molecule that is present in multiple copies per cell. mtDNA encodes proteins that are essential for ATP production through respiration [1]. mtDNA copy number is variable and responsive to changes in the level of ATP demand. Knowledge of the mechanisms underlying control of mtDNA copy number would enhance understanding of maintenance of cellular energy supply in response to various environment stresses. Failure to maintain mtDNA integrity affects cellular function, and is associated with aging as well as multiple human disorders including diabetes, cancer, and neurodegenerative disease [2], [3]. Thus, elucidation of the mechanisms that cause accumulation of mtDNA mutations will contribute to efforts to prevent mtDNA-related disorders.

DNA mutations can be acquired by inheritance, replication errors, and environmental damage such as nuclear radiation, ultraviolet light, or certain chemicals. The main cellular response to DNA damage is the nuclear checkpoint pathway, which arrests the cell cycle and gives the cell time to repair damage before replication and mitosis. This checkpoint pathway contains several protein kinases, including ataxia-telangiectasia mutated (ATM), and their downstream target Chk2, which are conserved in yeast and humans. Chk2 itself is a protein kinase, and is phosphorylated and activated in response to DNA damage [4]. In the budding yeast *Saccharomyces cerevisiae*, activation of Mec1/Rad53 (the yeast orthologs of human ATM/Chk2) up-regulates mtDNA copy number via augmentation of the deoxyribonucleoside triphosphate (dNTP) pools. This increase in copy number, which does not require the mtDNA replication pathway dependent on transcription factor Abf2 [5], is concomitant with accumulation of point mutations in mitochondrial genomes [6]. A recombination-mediated mtDNA replication pathway triggered by reactive oxygen species (ROS) exists in budding yeast [7]–[9]. Upon DNA damage in human cells, ATM activates the G2/M checkpoint through phosphorylation of Chk2; knockdown of ATM decreases mtDNA content [10], [11]. To date, however, no study has elucidated the mechanism(s) underlying such changes in mtDNA level in human cells. In addition, the effects of the ATM/Chk2 checkpoint pathway on mitochondrial genome integrity in human cells remain to be investigated.

**Figure 1:**
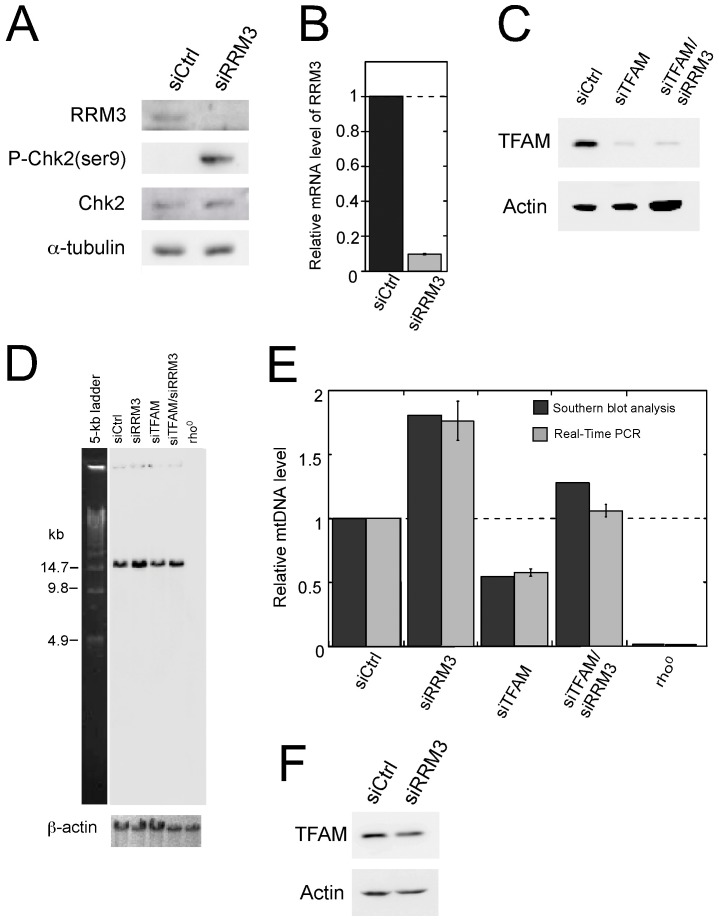
Effects of RRM3 knockdown on Chk2 phosphorylation and mtDNA content. (A) Detection of phosphorylated Chk2 kinase by Western blot analysis in RRM3 knockdown cells. (B) mRNA levels of RRM3 upon knockdown. Data are shown as the mean ± standard error of the mean (SEM) from three independent experiments. (C) Western blot analysis of proteins TFAM and Actin. Total cell lysates were extracted from TFAM knockdown cells and TFAM/RRM3 double-knockdown cells after transfection with the indicated siRNAs, and were analyzed using antibodies specific for TFAM and Actin (loading control). (D) Southern blot analysis of mtDNA level in cells in which RRM3 was knocked down, either alone or in combination with TFAM. Total cellular DNA was digested with *BamH*I, which has a single cutting site in human mtDNA. Signals of mtDNA and nuclear DNA were detected with probes corresponding to the OH region and β-actin, respectively. (E) Quantitative data of Southern blot and quantitative real-time PCR analyses of mtDNA level in cells in which RRM3 was knocked down, either alone or in combination with TFAM. Relative mtDNA level was calculated as the ratio of the signal from the OH region (representing mtDNA) to the signal from the **β-actin** gene (representing nuclear DNA), based on the results of Southern blot analysis in (D). Relative mtDNA level was also calculated as the ratio of the signal from the ND1 gene (representing mtDNA) to the signal from the **β-actin** gene (representing nuclear DNA) by quantitative real-time PCR. The average values plotted, based on quantitative real-time PCR, were obtained from three independent experiments. (F) Effect of RRM3 knockdown on TFAM level.

**Figure 2:**
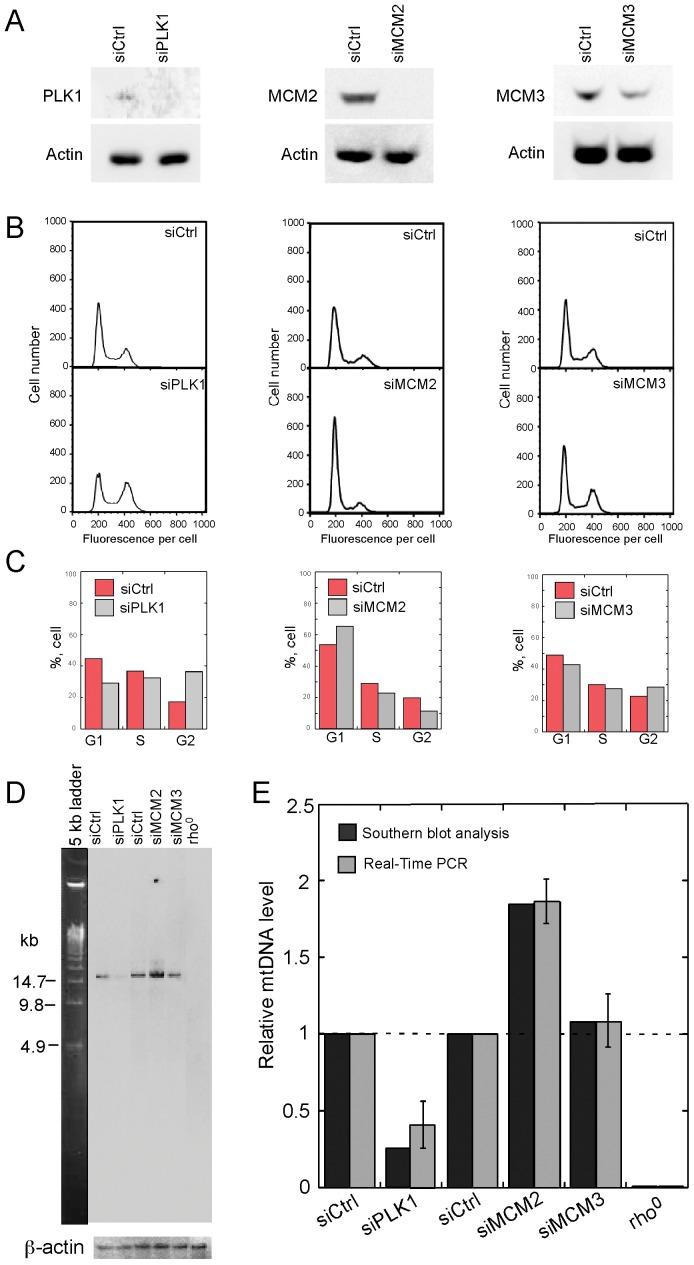
Effects of PLK1, MCM2, or MCM3 knockdown on mtDNA content. (A) Western blot analysis of proteins PLK1, MCM2, and MCM3. Total lysates in knockdown cells were extracted 24 h after transfection with siRNA against PLK1, or 72 h after transfection with siRNAs against MCM2 and MCM3, and analyzed using antibodies specific for PLK1, MCM2, MCM3, and Actin (loading control). (B) FACS analysis of DNA content in PLK1, MCM2, and MCM3 knockdown cells. DNA content was analyzed using flow cytometry 24 h after transfection with siRNA against PLK1, or 72 h after transfection with siRNAs against MCM2 and MCM3. (C) Percentage of cells at each phase during the cell cycle. (D) Southern blot analysis of mtDNA content in cells transfected with either control siRNA or the indicated siRNAs. Total cellular DNA in knockdown cells was extracted 24 h after transfection with siRNA against PLK1, or 72 h after transfection with siRNAs against MCM2 and MCM3, and digested with *BamH*I. Signals of mtDNA and nuclear DNA were detected with probes as described in Figure 1D. (E) **Quantitative analysis of relative** mtDNA levels obtained from Southern blot and real-time PCR analyses. Relative mtDNA levels were estimated by both Southern blot and quantitative real-time PCR analyses as described in Figure 1E. For real-time PCR analysis, data are shown as the mean ± standard error of the mean (SEM) from three independent experiments.

RRM3 is a 5'-3' DNA helicase that promotes replication fork progression. In *Saccharomyces cerevisiae*, disruption of RRM3 activates the Mec1/Rad53 checkpoint pathway [5] by inducing replication fork pauses at multiple sites throughout the ribosomal DNA during S phase [12].

In this study, we sought to determine whether depletion of human PIF1/RRM3 (an ortholog of yeast RRM3) also results in activation of the ATM/Chk2 checkpoint pathway via Chk2 phosphorylation and cell cycle arrest, leading to an increase in mtDNA copy number, as in budding yeast. We have observed that knockdown of RRM3 increases the level of Chk2 phosphorylation, indicating activation of the ATM/Chk2 checkpoint pathway. We further demonstrate that checkpoint activation increases mtDNA copy number independently of TFAM, and that ROS are required for this mtDNA increase. Furthermore, the mtDNA content increase is not due to delay of cell cycle progression, but likely relies on functions of cell cycle regulators related to mtDNA metabolism. Finally, we show that checkpoint activation induces the common 4977-bp mtDNA deletion. From these data, we infer that nuclear checkpoint activation influences mitochondrial genome integrity in human cells.

**Figure 3:**
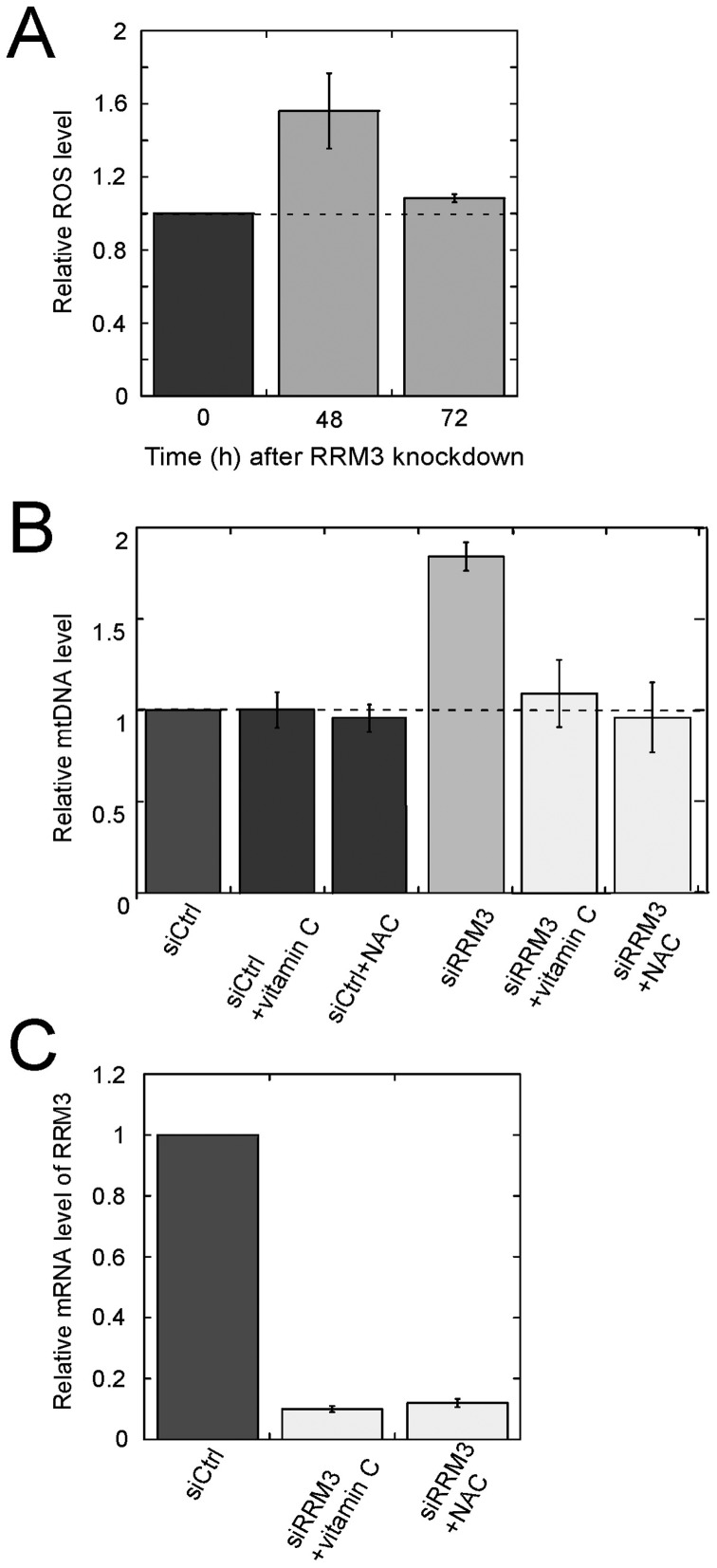
ROS are required for the increase in mtDNA content in RRM3 knockdown cells. (A) Relative ROS level. Cellular ROS generated were measured 48 h and 72 h after transfection with siRNA against RRM3, using the fluorescent probe CM-H2DCFDA. The relative ROS level was defined as the measured ROS level at 48 h and 72 h relative to the level at time 0, which was set to 1. (B) Quantitative real-time PCR analysis of mtDNA copy number in RRM3 knockdown cells. Total cellular DNA was isolated 72 h after transfection with siRNA against RRM3 in the absence or presence of the ROS scavengers N-acetylcysteine (NAC) or vitamin C. Relative mtDNA level was determined as described in Figure 1E for quantitative real-time PCR. (C) mRNA levels of RRM3 upon knockdown in the presence of either vitamin C or NAC. Data are shown as the mean ± standard error of the mean (SEM) from three independent experiments.

## Materials and Methods

### Cell Culture and Gene Knockdown

HeLa S3 cells (ATCC, CCL-2.2) were grown in DMEM (Invitrogen) containing 10% FBS and **penicillin/streptomycin (**100 units/ml and 100 µg/ml, respectively)**.** SiRNA duplexes targeted to RRM3 were designed as described [13] and synthesized by Dharmacon RNAi Technologies (USA), with the following sequences: sense, 5′- CCUCAGACCAGGAGAACAUUU-3′; antisense, 5′- AUGUUCUCCUGGUCUGAGGUU-3′. TFAM SMARTpool siRNA was purchased from Dharmacon. Stealth siRNA for PLK1, MCM2, and MCM3 were custom designed and synthesized by Invitrogen, with the following sequences: PLK1 sense, AGAAGACCCUGUGUGGGACUCCUAA; PLK1 antisense, UUAGGAGUCCCACACAGGGUCUUCU; MCM2 sense, GAGCUCAUUGGAGAUGGCAUGGAAA; MCM2 antisense, UUUCCAUGCCAUCUCCAAUGAGCUC; MCM3 sense, GGGAGCUGAUCAGUGACAACCAAUA; MCM3 antisense, UAUUGGUUGUCACUGAUCAGCUCCC. HeLa cells were transfected with either target siRNAs or a non-targeting negative control siRNA (Dharmacon), using either DharmaFECT 1 transfection reagent (Dharmacon) for Dharmacon siRNAs or LipofectamineTM RNAiMAX (Invitrogen) for Stealth siRNAs. Knockdown efficiency of RRM3 was confirmed by measuring mRNA levels using real-time RT-PCR and by Western blot analyses. Knockdown efficiency of TFAM, PLK1, MCM2, and MCM3 were confirmed by measuring protein levels via Western blot. Total RNA was isolated from control and knockdown HeLa cells 48 h after transfection using an RNeasy Mini kit (Qiagen). Reverse transcription of RNA was performed using a TaKaRa RNA PCR kit (AMV, ver.3.0, TaKaRa). The primers used for real-time PCR were as follows: RRM3-forward, 5′-GATGTGGCCCTCACCAAC-3′; RRM3-reverse, 5′-TGCTGTCCATAGCCTCAAATC-3′; **β-actin**-forward, 5′-ATGAAGATCAAGATCATTGCTCCTC-3′; **β**-actin-reverse, 5′-ACATCTGCTGGAAGGTGGACA-3′. The 20 µl reaction volumes contained 10 µl of SYBR Premix Ex Taq™ II (Takara), 0.4 µM of each primer, and <100 ng of template genomic DNA. A LightCycler 480 (Roche) was used for real-time PCR analysis.

**Figure 4:**
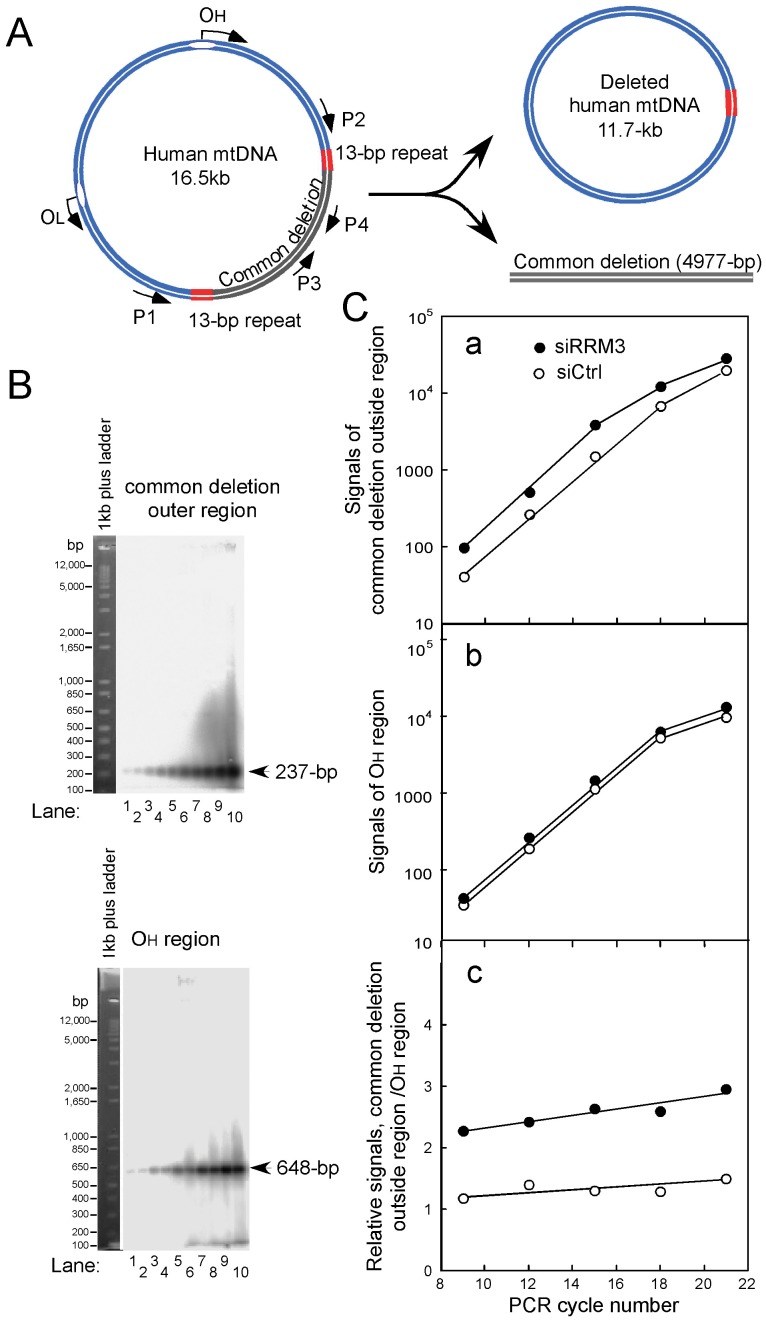
Effects of RRM3 knockdown on generation of the common 4977-bp deletion. (A) Locations of the designed primers on the mitochondrial genome. (B) Detection of the common deletion using quantitative PCR-based analysis. Total cellular DNA, used as a template for PCR, was extracted from control HeLa cells (lanes 1, 3, 5, 7, 9) or RRM3-knockdown HeLa cells (lanes 2, 4, 6, 8, 10). The P1 and P2 primers, which hybridize outside the deletion region, were used to detect the common 4977-bp deletion. This primer pair yields a 237-bp PCR product only if the deletion is present. PCRs were performed for 9, 12, 15, 18, and 21 cycles. PCR products were separated by electrophoresis on 2% agarose gels and detected by Southern blot analysis using ^32^P-labeled DNA fragments as probes representing either the common deletion outer region (237-bp; upper panel) or the OH region (648-bp; lower panel)**.** (C) Increased common 4977-bp mtDNA deletion upon RRM3 knockdown. The values of the signals corresponding to the common deletion outer region (a) and OH region (b) in (B), after background subtraction, were plotted as log graphs. The ratios of the common deletion outer region to the OH region at various PCR cycles were plotted (c).

**Figure 5:**
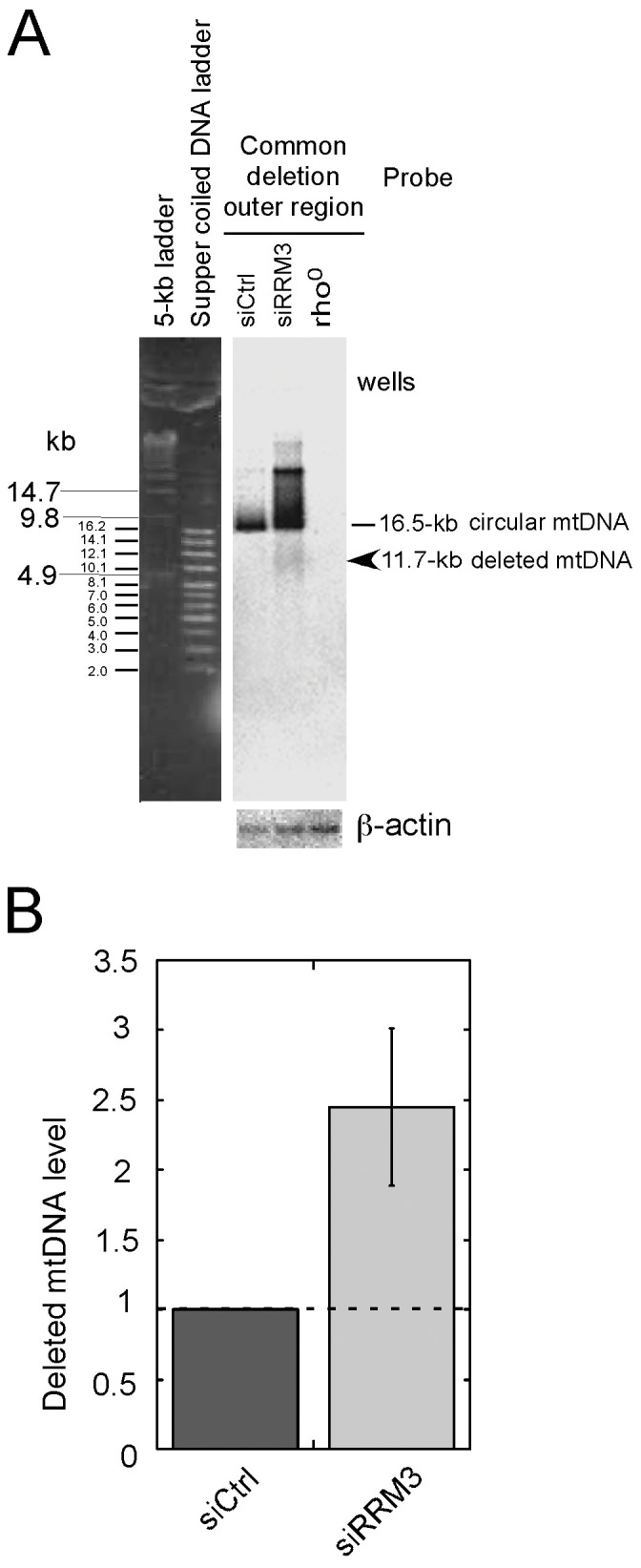
Direct detection of mtDNA lacking the 4977-bp region in RRM3-knockdown cells. (A) Detection of deleted mtDNA signals. Total cellular DNA (approximately 37.5 µg) extracted from control cells, RRM3 knockdown cells, and rho^0^ cells was separated by electrophoresis. DNA was transferred to membranes and hybridized with probes corresponding to the outer common deletion region (CD) (for detection of signals derived from mtDNA) or β-actin (for nuclear DNA signals). DNA size markers: (1) 5-kbp ladder (Bio-Rad) and (2) supercoiled DNA ladder (Promega). The outer CD region probe is designed to detect the normal 16.5-kb circular mtDNA and the deleted 11.7-kb circular mtDNA, if the deletion is present. (B) Quantitative analysis of mtDNA lacking the 4977-bp region. The levels of mtDNA lacking the 4977-bp region were estimated from the ratio of the 11.7-kb circular mtDNA (indicated by arrow) to nuclear DNA (represented by β-actin), and normalized to the value for control cells. Data are shown as the mean ± standard error of the mean (SEM) from five independent measurements (n = 5). Statistical significance of the difference as calculated by Student’s *t*-test is with p<0.002.

**Figure 6:**
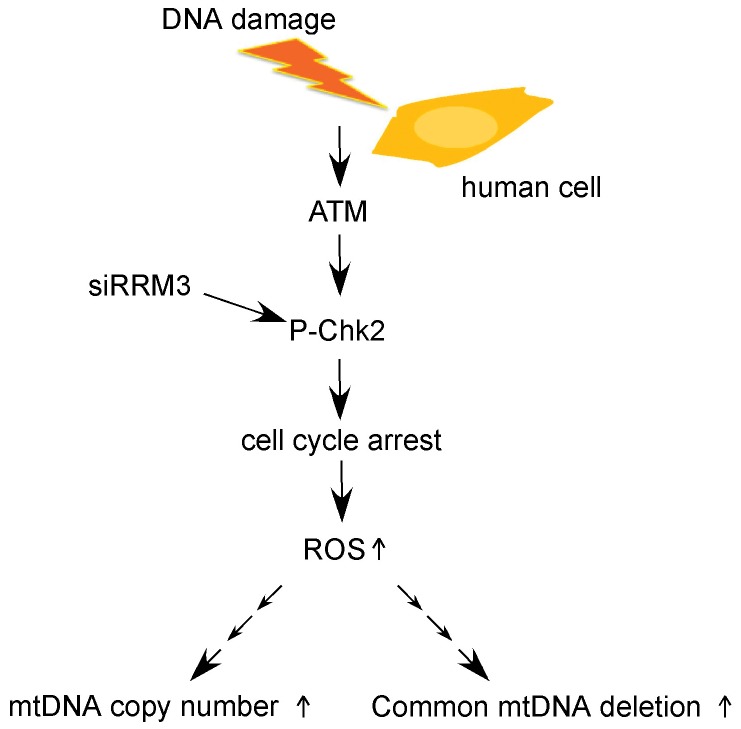
A pathway for the increases in mtDNA content and 4977-bp mtDNA deletion upon ATM/Chk2 checkpoint activation in human cells. Knockdown of RRM3 enhances phosphorylation of Chk2, indicating activation of the ATM/Chk2 checkpoint and delay of cell cycle progression. ROS level is significantly elevated. Checkpoint activation increases mtDNA copy number in a TFAM-independent but ROS-dependent manner. Concomitantly, the common 4977-bp mtDNA deletion is induced as a consequence of accumulation of oxidative mtDNA damage.

### Western Blot Analysis

Western blot analysis was performed using extracts obtained from control and knockdown cells 72 h after transfection (or 24 h for the PLK1 knockdown cells). Primary antibodies were anti-PIF1/RRM3 (sc-68870, 1∶250, Santa Cruz), anti-phospho-Chk2S19 (2666, 1∶1000, Cell Signaling), anti-Chk2 (2662, 1∶1000, Cell Signaling), anti-α-tubulin (B-5-1-2, 1∶5000, Sigma), anti-TFAM (ab89818, 1∶1000, abcam), anti-PLK1 (4535, 1∶500, Cell Signaling), anti-MCM2 (4007, 1∶500, Cell Signaling), anti-MCM3 (4012, 1∶500, Cell Signaling) and anti-Actin (MAB1501, 1∶1000, Chemicon).

#### Intracellular ROS detection

Intracellular ROS generation after RRM3 knockdown was detected by using the fluorescent probe CM-H2DCFDA (Molecular Probes). Transfected cells (1×10^5^/ml) were loaded with 10 µM CM-H2DCFDA and incubated for 30 min at 37°C. Aliquots of 100 µl were taken to measure the fluorescent products at an excitation wavelength of 488 nm and an emission wavelength of 520 nm in a SpectraMax fluorescent plate reader (Molecular Devices).

#### Fluorescence Activated Cell Sorting (FACS) analysis

Transected cells were trypsinized, fixed with cold methanol at −20°C for more than 3 h, and stained with 50 µg/ml propidium iodide and 5 µg/ml RNase A in PBS. DNA content was then analyzed by flow cytometry at RRC (Research Resources Center, RIKEN Brain Science Institute, Japan).

### DNA Extraction and Real-time PCR for mtDNA Copy Number Analysis

Total cellular DNA was extracted 72 h after transfection (or 24 h for the PLK1 knockdown cells) using DNAzol Reagent (Invitrogen). Relative mtDNA copy number was defined as the ratio of mtDNA (represented by the ND1 gene) to nuclear DNA (represented by the β-actin gene). The primers for the ND1 gene were: ND1-forward, 5′-CCCTAAAACCCGCCACATCT-3′; ND1-reverse, 5′-GAGCGATGGTGAGAGCTAAGGT-3′. The primers for the β-actin gene are as described above. Two independent reactions for mitochondrial and nuclear primer sets were run for each sample. DNA samples were diluted 10- to 40-fold; various dilutions of the template DNA were used in the PCR reaction to ensure measurements were within the linear range of the assay.

### Primers and Probes for Detection of the 4977-bp Common Deletion

Primers were designed for detection of the 4977-bp common deletion as described [14]. Common deletion outer region: P1, 5′-AAAATATTAAACACAAACTACCACCTACCTCCCTCACCAT-3′; P2, 5′-GGGGAAGCGAGGTTGACCTG-3′. Common deletion inside region: P3, 5′-CTGAGCCTTTTACCACTCCAG-3′; P4, 5′-GGTGATTGATACTCCTGATGCG-3′. OH region: forward, 5′- TAACCACTCACGGGAGCTCT-3′; reverse, 5′-AAGGCTAGGACCAAACCTAT-3′. β-actin gene (for nuclear DNA): β-actin-forward, 5′-TGCGTGACATTAAGGAGAAGCTGTGC-3′; β-actin-reverse, 5′-CTCGTCATACTCCTGCTTGCTGATCC-3′. Probes for detection of the 4977-bp deletion, mtDNA OH region, and nuclear DNA were amplified by PCR using the appropriate primer sets. The common deletion inner region probe was generated using the P3 and P4 primer pair, while the common deletion outer region probe was generated using the P1 and P2 primer pair.

### Southern Blot Analysis

Total cellular DNA (approximately 15 µg) was separated by electrophoresis on a 0.5% agarose gel, run at 4°C for 30 h at 1 V/cm, and transferred to a nylon membrane (Amersham Hybond N Plus; GE Healthcare). Signals for mtDNA were detected using the appropriate ^32^P-labeled mtDNA fragments as probes. In parallel, the same amount of total cellular DNA was separated by electrophoresis on a 1.0% agarose gel, run at 4°C for 2 h at 5 V/cm, and transferred to a membrane. Signals for nuclear DNA were detected using the appropriate ^32^P-labeled DNA fragment of β-actin as the probe. Signals corresponding to mtDNA and nuclear DNA fragments were quantitated using a Fuji BAS 2500 image analyzer as described [7].

## Results

### Activation of the ATM/Chk2 Checkpoint Pathway and up-regulation of mtDNA Copy Number Upon Knockdown of RRM3

In order to activate the ATM/Chk2 checkpoint pathway, we first knocked down the RRM3 gene in HeLa cells, using siRNA. Compared to ionizing radiation or other methods, which cause indiscriminate damage in both nuclear and mtDNA, activation of the ATM/Chk2 nuclear checkpoint pathway by knockdown of RRM3 allowed us to isolate the effect of the human nuclear DNA damage response system on mitochondrial genome homeostasis. The knockdown efficiency of RRM3 was confirmed by both Western blot (Fig. 1A) and RT-real time PCR (Fig. 1B). Using a phospho-Chk2 antibody, we observed significant phosphorylation of Chk2 at Ser-19, which is phosphorylated by ATM (Fig. 1A). Moreover, both Southern blot analysis and real-time PCR indicated that mtDNA copy number increased approximately 1.8-fold in the RRM3 knockdown cells compared to cells transfected with a scrambled control siRNA (Fig. 1D and 1E). These results indicate that in human cells, knockdown of RRM3 activates the ATM/Chk2 checkpoint pathway and increases mtDNA content.

### The Increase in mtDNA Copy Number during Checkpoint Activation is Independent of TFAM

We wished to determine whether the increase in mtDNA copy number upon RRM3 knockdown is dependent on TFAM (human mitochondrial transcription factor A), which is required for mtDNA replication and maintenance and is postulated to be a key regulator of mtDNA copy number. To this end, we knocked down TFAM, either alone or in combination with RRM3. Both Southern blot and real-time PCR analyses revealed that TFAM knockdown decreases mtDNA copy number to 55.5% of control levels; in contrast, mtDNA copy number was elevated above that of control cells when cells were simultaneously co-transfected with siRNAs against RRM3 and TFAM (Fig. 1D and 1E). Knockdown of TFAM was confirmed at the protein level via Western blot (Fig. 1C). We further revealed that checkpoint activation did not affect TFAM protein level (Figure 1F). Therefore, the increase in mtDNA copy number upon ATM/Chk2 checkpoint activation occurs independently of TFAM.

### The Increase in mtDNA Copy Number Upon ATM/Chk2 Checkpoint Activation is not Simply Due to a Delay in Cell Cycle Progression

To determine whether the increased mtDNA level upon ATM/Chk2 checkpoint activation is the consequence of a delay in cell cycle progression, we examined the changes in mtDNA level in cells transfected with siRNAs against several cell cycle regulators. Polo-like kinase 1 (PLK1) is required for functional maturation of the centrosome in late G2/earlyM prophase and establishment of the bipolar spindle; depletion of PLK1 causes G2/M cell-cycle arrest with 4*N* DNA content, and can induce apoptosis [15]. Minichromosome maintenance proteins (MCMs) are essential for the initiation of required DNA replication; depletion of MCMs results in cell cycle arrest [16]. We knocked down PLK1, MCM2, and MCM3 in HeLa cells using appropriate siRNAs. The knockdown efficiency of each gene was confirmed by Western blot analysis (Fig. 2A). Knockdown of PLK1 resulted in a significant increase in G2/M phase cells and a corresponding decrease in G1 cells 24 h after transfection. Knockdown of MCM2 resulted in a slightly increased G1 population and a corresponding decreased S population, whereas knockdown of MCM3 showed no significant change in cell cycle profile (Fig. 2B and 2C). All of the cell cycle profiles obtained in these knockdown experiments are coincident with previous observations [15], [16]. Contrary to our expectations, PLK1 depletion caused a remarkable reduction of mtDNA copy number (Fig. 2D and 2E). mtDNA copy number was unchanged in MCM3 knockdown cells, but increased approximately 1.8-fold in MCM2 knockdown cells (Fig. 2D and 2E). All of these data were double-checked using both Southern blot and real-time PCR analyses. Thus, the increase in mtDNA content upon ATM/Chk2 checkpoint activation is unlikely to be due to a delay in cell cycle progression.

### Involvement of ROS in Increase in mtDNA Copy Number Upon Knockdown of RRM3

Next, we sought to achieve mechanistic insight into the TFAM-independent mtDNA replication pathway activated by the ATM/Chk2 checkpoint. Since a ROS-triggered, recombination-mediated mtDNA replication pathway is known to exist in budding yeast, we hypothesized that a similar mechanism might be present in human mitochondria. If so, elevation of ROS above some threshold level would be required for the checkpoint activation-induced increase in mtDNA content. In order to test this hypothesis, we first monitored ROS levels after knockdown of RRM3. As shown in Figure 3A, 48 h after transfection with RRM3 siRNA, the ROS level was elevated approximately 1.5-fold relative to control; by 72 h after transfection, the ROS level had almost been restored to the same level as control cells. Next, we investigated whether the elevated ROS level is required for the increase in mtDNA copy number by transfecting HeLa cells with RRM3 siRNA in the presence of the ROS scavengers N-acetylcysteine (NAC) and vitamin C [9]. In the presence of NAC or Vitamin C, the increase in mtDNA content was completely suppressed (Fig. 3B); neither vitamin C nor NAC affects RRM3 knockdown efficiency at the mRNA level (Fig. 3C). Taken together, these results suggest that ROS are required for the checkpoint activation-induced increase in mtDNA content through a mtDNA replication pathway that is independent of TFAM.

### An Increase in the Frequency of the 4977-bp Common Deletion Upon Checkpoint Activation

To determine whether RRM3 knockdown affects mitochondrial genome integrity, we used two different techniques to detect the common 4977-bp deletion. We first performed quantitative PCR-based analysis using primers designed to amplify the 237-bp common deletion outer region and the 648-bp OH region (Fig. 4A). These signals were detected by quantitative Southern blot analysis (Fig. 4B and 4C). The frequency of the common 4977-bp deletion was estimated from the ratio of the level of the 237-bp common deletion outer region to the level of the OH region at different PCR cycles (Fig. 4B and 4C-a and -b). We detected an approximately 2.1-fold increase (mean value at different PCR cycles) in the common 4977-bp deletion in RRM3 knockdown cells, relative to control cells (Fig. 4C-c). Secondly, we attempted to detect the signals derived from linear DNA fragments of the common 4977-bp deletion via Southern blot analysis. The signals are faint (Fig. S1), probably because linear double-stranded DNA molecules are easily digested by mitochondrial exonucleases. Therefore, we further validated this observation by detecting a 2.5-fold increase in mtDNA molecules lacking the 4977-bp region (an 11.7-kb circular molecule) in RRM3 knockdown cells compared with control cells (Fig. 5A and 5B). Taken together, the results indicate that ATM/Chk2 checkpoint activation induces the common 4977-bp mtDNA deletion while concomitantly increasing mtDNA copy number.

## Discussion

In this study, we showed that knockdown of the RRM3 gene in human cells results in enhanced phosphorylation of Chk2, a TFAM-independent increase in mtDNA content, elevated ROS levels, and induction of the common 4977-bp mtDNA deletion (Fig. 6).

### “Why” and “How” of the Increase in mtDNA Copy Number Triggered by Checkpoint Activation

Our results show that checkpoint activation upon knockdown of the RRM3 gene up-regulates mtDNA copy number (Figs. 1D, 1E, and 3B). MtDNA replication occurs continuously, regardless of growth state, in both proliferating and post-mitotic cells [17]. Thus, it is reasonable to predict that the observed increase in mtDNA copy number results from continued mtDNA replication while the cell cycle is arrested by nuclear checkpoint control. Our results, however, reveal that mtDNA level varies among cells in which different cell cycle regulators have been knocked down. Therefore, it is unlikely that the increase in mtDNA content is simply due to continuous mtDNA replication upon cell-cycle arrest.

Increases in mtDNA content and mitochondrial proteins, followed by an increase in ATP synthesis by mitochondria, are important for cell cycle progression [18]. When DNA damage occurs inevitably, ATP is required for checkpoint activation accomplished by multiple kinases that catalyze the transfer of a phosphoryl group from ATP to an acceptor molecules, for DNA repair synthesis, as a precursor to dATP [19], and for other events (*e.g.*, DNA ligation catalyzed by ATP-dependent DNA ligases) when the checkpoint is activated. We postulate that the increased mtDNA copy number observed upon checkpoint activation is likely to represent a response to ATP demand; upregulation of mtDNA content allows more rapid synthesis of mtDNA-encoded proteins, which (along with nuclear DNA-encoded proteins) include necessary subunits of the electron transport chain complexes.

Our results show that checkpoint activation up-regulates mtDNA copy number even when the TFAM protein level is reduced (Fig. 1D and 1E), indicating that the increase in mtDNA content is independent of TFAM. Previous results demonstrated that despite mtDNA copy number increases from early G1/S to G2 phase, the level of TFAM, the transcriptional factor that regulates mtDNA copy number, does not change during this process [20]. Taken together, these results support the idea that a TFAM-independent replication pathway exists in human cells.

The enhanced ROS level upon RRM3 knockdown, and the repressive effect of ROS scavengers on the increase in mtDNA content upon checkpoint activation, have several implications (Fig. 3). Mitochondrial protein synthesis occurs mainly during S phase; mitochondria in S and G2/M phases contain significantly higher ROS levels relative to G1 phase [21]. Thus, ROS levels vary over the course of cell cycle progression. Our observation of higher ROS levels may provide some insights into the mechanism underlying TFAM-independent mtDNA replication. A link between increased mtDNA copy number and increased oxidative stress has been observed in human cells [11], [22]. Recently, a transitory increase in mtDNA copy number, accompanied by ROS generation, was observed after low-dose X-irradiation in a human breast cancer cell line [23]. These observations reminded us of ROS-triggered, mtDNA recombination-mediated replication in budding yeast [7], [8], which also contributes to mechanisms of yeast mtDNA maintenance that are dependent on mitochondrial fusion [9]. In budding yeast, Mhr1 catalyzes an important step of recombination termed homologous DNA pairing [24]. The 3′-single stranded DNA terminus in the heteroduplex formed by Mhr1 is assumed to serve as a primer in the initiation of rolling circle type replication. Such a 3′-single-stranded DNA terminus may result from the processing of ROS-induced double strand breaks at the replication origin (*e.g.*, *ori5*) by the 5′-3′ exonuclease activity. In human mitochondria, Flap endonuclease (Fen1) is a likely candidate for this exonuclease: it has 5′-3′ exonuclease activity, is DNA damage–inducible, and is localized to both mitochondria and nuclei [25], [26]. The levels of Fen1 protein in mitochondria should be investigated in both wild type and RRM3 knockdown cells. The increase in mtDNA content upon checkpoint activation can be explained if we posit the presence of ROS-triggered, recombination-mediated replication in human mitochondria. The conditions for this type of replication are likely to be satisfied by checkpoint activation in response to DNA damage. A number of studies have demonstrated the existence of human mtDNA recombination [27]–[31]. Whether ROS-triggered, recombination-mediated replication truly exists in human cells, and whether it occurs in response to increases in mtDNA copy number upon activation of the ATM/Chk2 checkpoint pathway, still remains to be resolved.

### Hypothesized Mechanism for Induction of the Common mtDNA Deletion Upon Checkpoint Activation

Another important observation of this study is the detection of an increase in the common 4977-bp mtDNA deletion upon checkpoint activation (Figs. 4 and 5). As noted, we detected enhanced ROS levels in RRM3 knockdown cells (Fig. 3); increased oxidative stress inside mitochondria may result in oxidative damage to mtDNA [32]. Since there was no obvious change in the level of TFAM, which protects against oxidative damage, accompanied by an increase of the mitochondrial genome upon checkpoint activation (Fig. 1F), we infer newly synthesized mtDNA molecules are highly susceptible. In addition, incorporation of pre-mutagenic damaged nucleotides, produced by ROS, into DNA also enhances mutagenesis in mitochondrial genomes [33], [34]. The common 4977-bp mtDNA deletion lies between two 13-bp direct repeats in the circular mtDNA molecule; the generation of this deletion by intra-molecular recombination most likely occurs during repair of damaged mtDNA [35]–[37].

Taken together, the data indicate that upon checkpoint activation, ROS are critical for the increase in mtDNA copy number; meanwhile, as a side effect, ROS cause mitochondrial mutagenesis, including the common 4977-bp mtDNA deletion.

Our study of checkpoint activation-induced increase in mtDNA copy number will contribute to understanding of the regulatory mechanisms underlying the dynamic regulation of mtDNA copy number, which is critical for maintenance of the cellular energy supply. Furthermore, the common 4977-bp deletion accumulates with age in normal individuals, primarily in non-dividing cells such as in muscle and brain tissue, and is also associated with various human disorders [38]–[40]. Thus, this work provides a unique insight into understanding the aging process, and provides clues that might aid in the development of new therapies against mtDNA-related disease.

## Supporting Information

Figure S1 **Direct detection of the 4977-bp region of mtDNA in RRM3-knockdown cells.** Total cellular DNA extracted from control cells, RRM3 knockdown cells, and rho^0^ cells was separated by electrophoresis. The DNA was transferred to membranes and hybridized with probes corresponding to the common deletion (CD) inner region or to β-actin. Lanes 1 and 2 are DNA size markers: (1) lambda/*Hind* III (NEB), and (2) 5-kbp ladder (Bio-Rad). The CD inner region probe is designed to detect the normal 16.5-kb circular mtDNA and the 4977-bp linear DNA fragment if the deletion is present. White and black arrowheads indicate the position of the 4977-bp linear DNA fragment.
(DOC)
