# Supplementary material for: Increases in Mitochondrial DNA Content and 4977-bp Deletion upon ATM/Chk2 Checkpoint Activation in HeLa Cells
Source: PLoS One. 2012 Jul 10;7(7):e40572. doi: 10.1371/journal.pone.0040572 (PMC3393681; doi:10.1371/journal.pone.0040572)
Supplement: Figure S1 — Direct detection of the 4977-bp region of mtDNA in RRM3-knockdown cells. Total cellular DNA extracted from control cells, RRM3 knockdown cells, and rho0 cells was separated by electrophoresis. The DNA was transferred to membranes and hybridized with probes corresponding to the common deletion (CD) inner region or to β-actin. Lanes 1 and 2 are DNA size markers: (1) lambda/Hind III (NEB), and (2) 5-kbp ladder (Bio-Rad). The CD inner region probe is designed to detect the normal 16.5-kb circular mtDNA and the 4977-bp linear DNA fragment if the deletion is present. White and black arrowheads indicate the position of the 4977-bp linear DNA fragment. (DOC) [file pone.0040572.s001.doc]

Niu *et al.*, Supplementary Information *PLoS ONE*

**Increases in Mitochondrial DNA Content and 4977-bp Deletion Upon ATM/Chk2 Checkpoint Activation in HeLa Cells**

Rong Niu ^1, 2^, Minoru Yoshida^1, 2^ and Feng Ling^1, 2^, *

^1^Chemical Genetics Laboratory, RIKEN Advanced Science Institute, ^2^JST-CREST

Hirosawa 2-1, Wako-shi, Saitama 351-0198, Japan.

# Supplementary Figure 1.

#
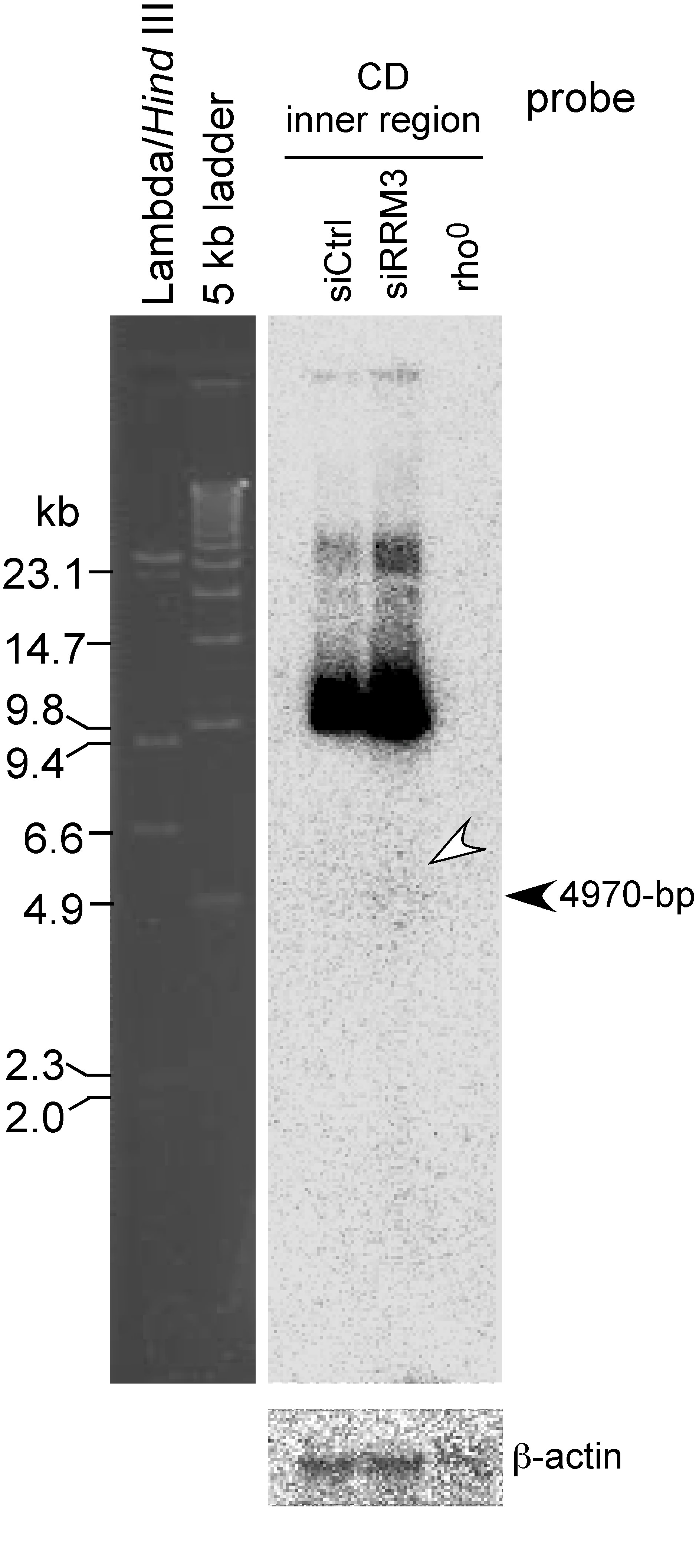
**Direct detection of the 4977-bp region of mtDNA in RRM3-knockdown cells.**

Total cellular DNA extracted from control cells, RRM3 knockdown cells, and rho^0^ cells was separated by electrophoresis. The DNA was transferred to membranes and hybridized with probes corresponding to the common deletion (CD) inner region or to β-actin. Lanes 1 and 2 are DNA size markers: (1) lambda/*Hind* III (NEB), and (2) 5-kbp ladder (Bio-Rad). The CD inner region probe is designed to detect the normal 16.5-kb circular mtDNA and the 4977-bp linear DNA fragment if the deletion is present. White and black arrowheads indicate the position of the 4977-bp linear DNA fragment.
